# Influence of printing angle on the mechanical and surface properties of 3D-printed hybrid resins for indirect restorations: An in vitro study

**DOI:** 10.4317/jced.64422

**Published:** 2026-08-28

**Authors:** Laura Buarque Caminha Lins, Pedro Thiago de Oliveira Neves, Anny Caroline Rodrigues Acioli, Ana Luisa Cassiano Alves Bezerra, Bruno Gustavo da Silva Casado, Rafaella de Souza Leão

**Affiliations:** 1 University of Pernambuco (UPE). Recife, Pernambuco, Brazil

**Keywords:** Printing, three-dimensional, materials testing, dental materials.

## Abstract

**Background:**

3D-printed hybrid resins are promising. However, some parameters, such as the printing angle, may affect their performance, and the available evidence is still limited. The purpose of this study was to evaluate the influence of different printing angles (0º, 45º, and 90º) on the mechanical and surface properties of 3D-printed hybrid resins.

**Materials and Methods:**

Groups were defined by printing angle (0°, 45°, 90°) and two resin types: VarseoSmile Crown Plus (VS) and PriZma Bio Crown (PB). Tests included flexural strength (n=10), surface roughness (n=12), microhardness (n=12), and contact angle (n=12). Statistical analyses compared materials (t-tests or Mann-Whitney) and printing angles (ANOVA or Kruskal-Wallis).

**Results:**

Flexural strength varied only in VS, with 90° showing the best results. Roughness was influenced in all groups, with 0° producing the lowest values. For microhardness, VS performed best at 90° and 45°, while PB performed best at 0° and 90°. Contact angle differences appeared only in VS, with 45° showing the greatest wettability.

**Conclusions:**

The printing angle affected most properties. A 0° angle yielded the best surface roughness for all materials and the best microhardness for PB. A 45° angle showed a favorable contact angle. A 90° angle was most suitable for flexural strength.

## Introduction

Within the digital workflow, additive manufacturing represents a process that generates less material waste and requires lower infrastructure costs compared to subtractive manufacturing [1-8]. Additionally, it enables the fabrication of complex geometries, including areas inaccessible to milling burs [9]. The designed object is produced by the sequential deposition of layers of liquid resin that undergo a light-curing process, typically performed through two main technologies: stereolithography (SLA) and digital light projection (DLP) [10,11].

Regardless of the light source employed for polymerization, the process is influenced by adjustable parameters generally specified by the manufacturers, such as layer thickness, degree of polymerization, post-curing time, and printing direction or angle [12,13]. The printing angle determines the orientation of the layers during manufacturing and significantly affects the accuracy and physical properties of the printed object; given the anisotropic nature of the material, its properties vary according to the direction of analysis [14-16]. This parameter is also commonly modified to optimize printing time or improve the distribution of objects on the printing platform [13].

It is essential that parts fabricated by additive manufacturing exhibit adequate resistance to environmental and mechanical stresses within the oral environment [17]. To enhance biological, physical, aesthetic, and mechanical performance, new hybrid materials have recently been introduced for additive manufacturing. In these formulations, ceramic particles and nanoparticles are incorporated into the resin matrix, combining the advantages of ceramics and resins to promote greater material longevity [18-19].

A recent systematic review [20] sought to determine a consensus regarding the optimal printing angle for 3D-printed resins. However, the findings revealed a gap in the literature, as available evidence was limited to flexural strength and marginal or internal fit properties. Moreover, none of the included studies addressed the behavior of hybrid resin compositions under different printing angles. A subsequent *in vitro* study [21] evaluated a 3D-printed nanoceramic hybrid resin fabricated at printing angles of 0°, 45°, and 90°, analyzing the degree of conversion, flexural strength, flexural modulus, surface roughness, and fractographic characteristics. Although this study represented a significant advance in understanding the influence of printing orientation on hybrid materials, it investigated only one commercial resin and did not evaluate other properties relevant to the clinical performance of these materials, such as surface wettability and microhardness.

Considering recent material innovations and the scarcity of data on this topic, the present study aims to evaluate the influence of different printing angles (0°, 45°, and 90°) on the mechanical and surface properties of hybrid printing resins, critical aspects when assessing the longevity and quality of restorations fabricated using this technique. The null hypothesis states that no significant differences exist among the evaluated printing angles.

## Materials and Methods

The specimens were divided into six groups according to three printing angles (0°, 45°, and 90°) and the two resins used: VarseoSmile Crown Plus- VS (Bego, Bremen, Germany) and PriZma Bio Crown- PB (MakertechLabs, São Carlos, SP, Brazil).

Prior to defining the final sample size, a pilot study was conducted with 10 specimens. The sample calculation was performed using the OpenEpi website (Open Source Epidemiologic Statistics for Public Health; Emory University, Atlanta, GA, USA. Available at: https://www.openepi.com). For this analysis, the mean and standard deviation values obtained in the flexural strength test for groups VS0 (50.49 ± 1.80), VS45 (48.91 ± 2.37), and VS90 (54.13 ± 1.28) were used. A two-sample t-test was applied considering a 5% margin of error (α=0.05), a 95% confidence interval, and a statistical power of 0.80, resulting in a required sample of 10 specimens per group. To account for potential losses during specimen preparation, the final sample size was set at 12 specimens per group for the surface roughness, contact angle, and microhardness analyses, and 10 specimens per group for the flexural strength test. Accordingly, disc-shaped specimens were fabricated for the surface property evaluations, totaling 72 specimens, and bar-shaped specimens were fabricated for the flexural strength test, totaling 60 specimens.

The specimens were designed using Meshmixer software, in disc (15 × 2.5 mm) and bar (25 × 2 × 2 mm) shapes, and were printed using a 3D printer (PioNext DJ-89; PioNext, Shenzhen, China) with previously calibrated printing parameters and designed according to the investigated printing angles (0º, 45º, and 90º) (Figure 1). After printing, post-processing was performed using a washing and post-curing device (Creality 3D UW-01; Creality, Shenzhen, Guangdong, China), according to the manufacturer's instructions. To regularize the surface, the specimens underwent finishing using sandpaper with decreasing grit sizes (360, 600, and 1200) and a metal device to guide wear. Then, all specimens were cleaned in an ultrasonic bath with distilled water for 5 minutes, with agitation to remove residues left by the grinding process.

To perform the three-point bending test, a mini universal testing machine (Microtensile OM150; ODEME, Luzerna, SC, Brazil) with a 5 kN load cell was used. The specimens (n=10) were placed in a horizontal position, in contact with two supports, each at a 60º angle, parallel to each other, at a distance of 20 mm between them. The force was applied to the center of the specimen by the machine's moving press at a constant speed of 1 mm/min. Finally, the flexural strength (MPa) was calculated using the formula S=3FL/2bd², where F is the fracture load (N) of the specimen, L is the width of the specimens (25 mm), and b and d are the width and thickness of the specimen, respectively.

The average surface roughness (Ra) was measured with a portable roughness tester (SURFTEST SJ-310; Mitutoyo Corporation, Kawasaki, Kanagawa, Japan) (n=12). Four measurements were performed in different directions, where the tip traveled a distance of 12 mm at a speed of 0.5 mm/s with a load of 0.7 mN. 

Microhardness was assessed using a Vickers hardness tester (ISH-MR 150; INSIZE, Suzhou, Jiangsu, China). Three indentations were made in the central portion of each specimen with a pyramid-shaped diamond indenter on the top surface of the specimens, and a 50 g load was applied for 20 seconds. The Vickers microhardness (HV) value was the average of the three measurements for each specimen. The HV was calculated by the device by dividing the applied load (F) by the impression area (A) left on the specimen, expressed by the equation: HV = F/A. The right and left static contact angles of a 2.0 μL distilled water droplet, measured using a volumetric pipette, were captured with a camera positioned 30 cm from the sample, 5 s after the droplet contacted the central area on both sides of each specimen. The contact angles were then measured by photographic analysis using the sessile drop method with ImageJ software (ImageJ 1.53e; National Institutes of Health, Bethesda, MD, USA). From the image, the software determined the contact angle value based on the droplet height and width measurements. Each specimen was measured twice, with different droplets.

Normality was verified using the Shapiro-Wilk test, and equality of variances was verified using Levene's F test. The margin of error used in the statistical tests was 5%. The data were presented using the following statistics: mean, standard deviation (mean ± SD), median, and 25th and 75th percentiles (median (P25; P75). For comparison between the two materials, the following statistical tests were used: Student's t-test with equal variances, Student's t-test with unequal variances, and Mann-Whitney. For comparison between angles, the F (ANOVA) or Kruskal-Wallis tests were used. In case of a significant difference by F (ANOVA), Tukey's or Tamhane's multiple comparison tests (between pairs of angles) were used. If the difference was verified by the Kruskal-Wallis test, Conover's multiple comparison test was used.

## Results

When comparing the printing angles regarding flexural strength, only the VS90 group showed a significant difference, exhibiting the highest mean value (*p<*0.001), as shown in Table 1. When comparing the materials, values were higher for VS than for PB (*p<*0.001) at all printing angles.

The results presented in Table 2 describe the surface roughness findings. For this outcome, the lowest values were observed at the 0° printing angle (*p<*0.001), and the best result was obtained for the VS0 group, with a value of 0.28 ± 0.06 (*p<*0.001).

For microhardness, the highest values were observed at the 90° printing angle, with VS showing statistically superior performance in all groups compared with PB. The highest mean value was recorded for VS90, with a value of 29.71 ± 4.20 (Table 3).

As shown in Table 4, only VS showed a significant difference among the printing angles evaluated for contact angle and wettability. The difference occurred between the VS45 group and the other two groups, with the VS45 group presenting the highest contact angle (*p<*0.001). When comparing the tested materials, significant differences were observed between the VS and PB groups at the 45° (*p<*0.001) and 90° (*p=*0.026) printing angles.

## Discussion

According to the results of this study, the printing angle showed a significant effect on the flexural strength of the VS material; therefore, the null hypothesis was rejected. These results are consistent with a previous study [21], which evaluated a hybrid resin printed at the same three orientations (0°, 45°, and 90°) and also observed that the printing angle significantly influenced flexural strength and flexural modulus. The 90° angle presented the highest values in the flexural strength test. These results are consistent with findings from a previous study [22], which also suggested a 90° printing angle. Similarly, another study [23] that tested VS resin concluded that specimens printed at a 90° angle exhibited greater flexural strength than those in the 0° and 45° groups.

A previous study [24] suggested that a 0° angle could be ideal for increasing strength, as it would allow the applied force to act perpendicular to the printing layers. However, the results of the present study indicate the opposite, as printing at a 90° angle causes the force to act parallel to the layer build direction. This concept is supported by other findings in the literature [22,23] and may be explained by additional factors, such as interlayer adhesion. Mudhaffer *et al*. [23] reported that the tested specimens did not exhibit distinct layers at the fracture sites, confirming homogeneity between layers, a condition that likely does not influence flexural strength. Furthermore, the degree of conversion of the material during polymerization should be considered. It is assumed that the 90° angle requires a greater number of layers to construct the object, resulting in longer light exposure and a higher degree of conversion, which may positively affect flexural strength.

Additionally, according to ISO 4049, 100 MPa is the minimum standard required for flexural strength in resin-based materials. In the present study, none of the investigated groups achieved this standard, highlighting the need for improvements in these materials from a mechanical standpoint, as fracture remains one of the main causes of clinical failure in definitive restorations. From a clinical perspective, these findings may indicate potential limitations regarding the long-term performance of 3D-printed hybrid resins, particularly in stress-bearing areas subjected to repeated occlusal forces. Although these materials offer advantages related to digital manufacturing workflows, improvements in their mechanical properties are still required to ensure greater reliability for definitive restorative applications.

From a surface characteristics perspective, low roughness values are known to positively influence the behavior of restorative materials in the oral environment, as smoother surfaces reduce biofilm accumulation and help preserve periodontal health [25]. This study demonstrated that printing angulation significantly affected the surface roughness of the specimens, also leading to the rejection of the null hypothesis. The 0° angle, represented by the VS0 group, was the only one that presented values close to 0.2 µm, which is considered an acceptable roughness threshold for these materials [26]. 

The present roughness findings agree with those of a previous study [21], which also reported lower roughness values at the 0° printing angle. The 0° angle may yield lower roughness values, possibly due to the gradual connection between printing layers. Conversely, the 45° angle may result in higher roughness values because the diagonal layer arrangement can create step-like irregularities and rougher surfaces, thereby increasing overall roughness [27]. It should be noted that, in the present study, testing was performed on the unpolished surface of the samples, which may have contributed to the higher roughness values observed. The printing angle is known to influence roughness, particularly on unpolished surfaces, as observed in the analyzed specimens [25]. Polishing can remove surface irregularities caused by the staircase effect in inclined printing orientations; therefore, it can be assumed that, if these outer surfaces were polished, the differences related to printing angles might be minimized [25].

Regarding microhardness, the results indicate that the printing angle produced statistically significant differences. For both materials, the 90° angle presented the highest values, thereby rejecting the null hypothesis for this property as well. The literature provides limited data on the relationship between printing angle and microhardness values. Nonetheless, it has been suggested that the adhesion strength between printed layers is lower than the intrinsic strength of each layer [28]. However, the present findings contradict this assumption, since the 90° angle exhibited the best results, differing statistically from the 0° group, which may indicate strong interlayer adhesion. Furthermore, previous studies have reported that printing angle may not affect microhardness values [13]; this statement partially supported by the current results, as the 90° angle only differed significantly from the 0° group but was statistically similar to 45°. This reinforces the notion that reduced microhardness values are not necessarily associated with inclined build directions.

The contact angle is directly related to surface wettability, which determines whether a surface is hydrophilic or hydrophobic, providing information about surface heterogeneity and indicating the ability of saliva to spread over the material [29]. According to the findings, only the VS group showed surface energy affected by the printing angle, also leading to rejection of the null hypothesis. Within this group, the 45° angle exhibited the highest contact angle values, with an average of 91.71°. According to the literature [29] a contact angle above 65° characterizes a hydrophobic surface, as observed in all groups investigated in this study. A hydrophobic surface demonstrates higher resistance to liquids and decreased wettability, which can reduce bacterial adhesion by limiting the interaction between bacteria and the solid surface [2]. 

Both materials tested showed significant differences, with the VS resin demonstrating superior overall performance. When comparing their compositions, VarseoSmile Crown Plus resin contains 52% inorganic filler, whereas PriZma Biocrown contains only 14%. This suggests that higher inorganic filler content may improve the properties of 3D-printed resins. This assumption is supported by the literature, which reports that the addition of ceramic particles and nanoparticles can significantly enhance several properties, including flexural strength, fracture toughness, hardness, wear resistance, and thermal stability [30]. It is important to emphasize, however, that this study presents inherent limitations due to its *in vitro* design, which does not fully reproduce the complex conditions found in the oral environment.

## Conclusions

Based on the findings of this study, it can be concluded that the printing angle significantly influenced the evaluated properties in most groups. The 0° angle provided the best results for surface roughness across all materials and for microhardness in the PB material. The 45° angle yielded favorable outcomes for contact angle in the VS material. Finally, the 90° angle proved to be the most suitable orientation for achieving the highest flexural strength values. 

## Figures and Tables

**Table 1:** Evaluation of flexural strength according to angle and material (MPa).

	**Material**		
**Angle**	**PB Mean ± DP**	**VS Mean ± DP**	*P value*
0	35,18 ± 3,05	50,49 ± 1,80^(A)^	*p*^(1)^*<*0,001*
45	34,50 ± 1,35	48,91 ± 2,37^(B)^	*p*^(1)^*<*0,001*
90	35,67 ± 2,49	54,13 ± 1,28^(C)^	*p*^(1)^*<*0,001*
*P value*	*p*^(2)^*=*0,167	*p*^(2)^*<*0,001*	

(*) Significant difference at the 5.0% level. (1) Mann-Whitney test. (2) Kruskal-Wallis test with Conover multiple comparisons. If the letters in parentheses are different, a significant difference is confirmed between the corresponding angles.

**Table 2:** Evaluation of roughness according to angle and material (µm).

	**Material**		
**Angle**	**PB Mean ± DP**	**VS Mean ± DP**	*P value*
0	0,75 ± 0,31^(A)^	0,28 ± 0,06^(A)^	*p*^(1)^*<*0,001*
45	1,36 ± 0,39^(B)^	1,54 ± 0,28^(B)^	*p*^(2)^*=*0,209
90	0,96 ± 0,16^(C)^	0,83 ± 0,21^(C)^	*p*^(2)^*=*0,102
*P value*	*p*^(3)^*<*0,001*	*p*^(4)^*<*0,001*	

(*) Significant difference at the 5.0% level. (1) Mann-Whitney test. (2) Student's t-test with equal variances. (3) Kruskal-Wallis test with Conover multiple comparisons. (4) F-test (ANOVA) with Tamhane multiple comparisons. If the letters in parentheses are different, a significant difference is confirmed between the corresponding angles.

**Table 3:** Evaluation of microhardness according to angle and material (kgf/mm^2^).

	**Material**		
**Angle**	**PB Mean ± DP**	**VS Mean ± DP**	*P value*
0	13,22 ± 3,79^(A)^	25,05 ± 4,17^(A)^	*p*^(1)^*<*0,001*
45	10,88 ± 3,83^(B)^	28,79 ± 5,22^(AB)^	*p*^(1)^*<*0,001*
90	16,81 ± 4,94^(A)^	29,71 ± 4,20^(B)^	*p*^(2)^*<*0,001*
*P value*	*p*^(3)^*=*0,006*	*p*^(4)^*=*0,041*	

(*) Significant difference at the 5.0% level. (1) Mann-Whitney test. (2) Student's t-test with equal variances. (3) Kruskal-Wallis test with Conover multiple comparisons. (4) F-test (ANOVA) with Tukey multiple comparisons. If all letters in parentheses are distinct, a significant difference is confirmed between the corresponding angles.

**Table 4:** Evaluation of the contact angle according to the angle and the material.

	**Material**		
**Angle**	**Mean ± DP**	**Mean ± DP**	*P value*
0	82,63º ± 3,59	84,47º ± 4,00^(A)^	*p*^(1)^*=*0,213
45	78,44º ± 7,60	91,71º ± 3,63^(B)^	*p*^(2)^*<*0,001*
90	81,34º ± 3,50	84,61º ± 3,20^(A)^	*p*^(3)^*=*0,026*
*P value*	*p*^(4)^*=*0,222	*p*^(5)^*<*0,001*	

(*) Significant difference at the 5.0% level. (1) Mann-Whitney test. (2) Student's t-test with unequal variances. (3) Student's t-test with equal variances. (4) Kruskal-Wallis test. (5) F-test (ANOVA) with Tukey's multiple comparisons. If the letters in parentheses are different, a significant difference is confirmed between the corresponding angles.

**Figure 1:**
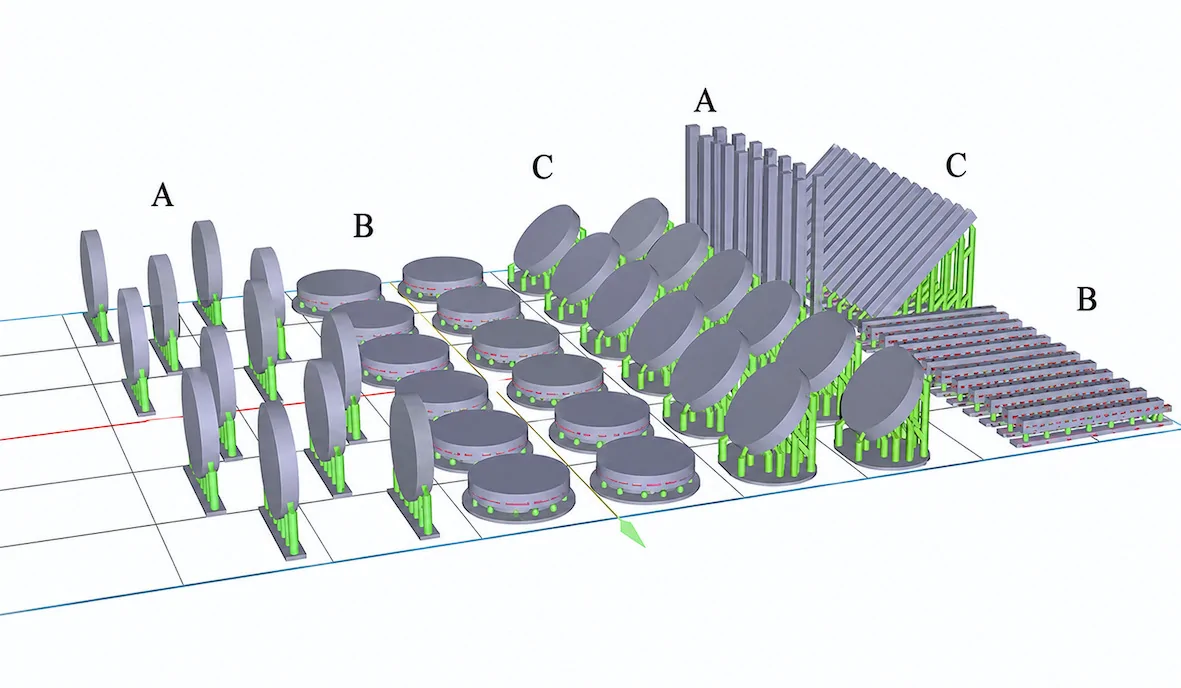
Design of specimens at the three angles: 90º (A), 0º (B), 45º (C).

## Data Availability

Declared none.
